# Genetic background influences age-related decline in visual and nonvisual retinal responses, circadian rhythms, and sleep^☆^

**DOI:** 10.1016/j.neurobiolaging.2014.07.040

**Published:** 2015-01

**Authors:** Gareth Banks, Ines Heise, Becky Starbuck, Tamzin Osborne, Laura Wisby, Paul Potter, Ian J. Jackson, Russell G. Foster, Stuart N. Peirson, Patrick M. Nolan

**Affiliations:** a MRC Harwell, Harwell Science and Innovation Campus, Oxfordshire, UK; b MRC Human Genetics Unit, MRC IGMM, University of Edinburgh, Western General Hospital, Edinburgh, UK; c Nuffield Laboratory of Ophthalmology (Nuffield Department of Clinical Neurosciences), University of Oxford, John Radcliffe Hospital, Oxford, UK

**Keywords:** Aging, Circadian, Sleep, Light input, Mouse strain

## Abstract

The circadian system is entrained to the environmental light/dark cycle via retinal photoreceptors and regulates numerous aspects of physiology and behavior, including sleep. These processes are all key factors in healthy aging showing a gradual decline with age. Despite their importance, the exact mechanisms underlying this decline are yet to be fully understood. One of the most effective tools we have to understand the genetic factors underlying these processes are genetically inbred mouse strains. The most commonly used reference mouse strain is C57BL/6J, but recently, resources such as the International Knockout Mouse Consortium have started producing large numbers of mouse mutant lines on a pure genetic background, C57BL/6N. Considering the substantial genetic diversity between mouse strains we expect there to be phenotypic differences, including differential effects of aging, in these and other strains. Such differences need to be characterized not only to establish how different mouse strains may model the aging process but also to understand how genetic background might modify age-related phenotypes. To ascertain the effects of aging on sleep/wake behavior, circadian rhythms, and light input and whether these effects are mouse strain-dependent, we have screened C57BL/6J, C57BL/6N, C3H-HeH, and C3H-Pde6b+ mouse strains at 5 ages throughout their life span. Our data show that sleep, circadian, and light input parameters are all disrupted by the aging process. Moreover, we have cataloged a number of strain-specific aging effects, including the rate of cataract development, decline in the pupillary light response, and changes in sleep fragmentation and the proportion of time spent asleep.

## 1 Introduction

In healthy individuals, a rhythmic sleep/wake cycle is maintained through the interaction of homeostatic and circadian mechanisms, as well as being modulated by external cues. The homeostatic process refers to an increase of sleep pressure that accumulates during wakefulness and is relieved by sleep (Borbely, 1982). The timing of the sleep/wake cycle is also regulated by the internal circadian clock, which provides an innate biological rhythm exerting control over a wide range of physiological and behavioral processes. The circadian clock is defined by its ability to maintain free running rhythms in the absence of external timing cues. However, to be of use, this clock must be synchronized to external cues—a process known as entrainment. In mammals, the most influential external cue for both sleep and circadian rhythms is light. Light is detected by the retina and, as well as its familiar role in image-forming vision, also acts to entrain circadian rhythms and directly modulate sleep by promoting sleep or alertness (Hubbard et al., 2013). Although the interactions between retinal light input pathways, the circadian clock, and the sleep homeostat are well maintained in healthy individuals, aging is known to have a negative impact on all these processes, leading ultimately to disrupted circadian rhythms and sleep/wake cycles in older individuals. In humans, the reported effects of aging include an increased occurrence of cataracts (Klein and Klein, 2013), loss of retinal photoreceptors (Gao and Hollyfield, 1992), reduced circadian regulation of melatonin and temperature (Pandi-Perumal et al., 2005, Weinert, 2010), a reduction in sleep duration and consolidation, and an increased susceptibility to misalignments in circadian phase (Dijk et al., 1999) and increased fragmentation of sleep and time spent asleep during the day (Huang et al., 2002).

Animal models have been used widely to understand the mechanisms underlying not only rhythmic behavior but also aging. Prominent among these models are inbred mouse strains. Inbred mice are commonly used in a range of biological research areas as their genetic homogeneity allows researchers in different laboratories to independently replicate results without the genetic background of the model being a confounding factor. They are also used extensively in genetic studies in which specific genes can be selectively knocked out or mutated to study their effects on the whole organism. A large number of different inbred mouse strains currently exist and through a combination of spontaneous mutation and genetic drift each inbred strain carries its own combination of mutations within its genome (Stevens et al., 2007). Two of the most commonly used mouse strains are C57BL/6J and C3H-HeH. The C57BL/6J strain has been used to characterize the influence of aging on both circadian rhythms and sleep (Hasan et al., 2012, Possidente et al., 1995, Valentinuzzi et al., 1997), whereas the C3H-HeH strain has been used in studies into how aging alters light inputs (Lupi et al., 2012, Semo et al., 2003a, Semo et al., 2003b).

Until recently, phenotypic analyses of most of the mouse genetic knockout models have been carried out on undefined mixtures of C57BL/6J and 129S7 mouse backgrounds. It is notable that a lack of consideration of the influence of these 2 strains on a phenotype has lead to confounding results. For example, initial studies using a mouse model of Fragile X syndrome on a mixed C57BL/6-129 background reported only mild learning deficits (D’Hooge et al., 1997). However, later work demonstrated that the influence of C57BL/6 could rescue a more severe learning deficit found using the same mice line on a 129 background (Dobkin et al., 2000). The latest mouse knockout resources such as the International Knockout Mouse Consortium are now producing knockout mouse models exclusively on the C57BL/6N background. Therefore, in the future most of the mouse phenotyping studies will use the C57BL/6N background rather than C57BL/6J or 129S7. The C57BL/6J and C57BL/6N strains have been separated by around 220 generations, and a comprehensive genotype comparison has demonstrated significant genetic differences between the 2 lines (Simon et al., 2013). Given these differences and the prominent use of C57BL/6N animals in knockout studies there is a requirement for baseline phenotyping and longitudinal studies that establish how this strain can be used as an animal model and exactly what phenotypic differences are evident among these 2 substrains. To date, differences have been reported between C57BL/6J and C57BL/6N in locomotor activity, anxiety measures, prepulse inhibition of acoustic startle response, grip strength, motor learning, and visual acuity (Matsuo et al., 2010, Simon et al., 2013). However, longitudinal studies exploring circadian rhythms, light responsiveness, and sleep activity differences between the 2 substrains have yet to be determined.

As noted previously, the C3H-HeH strain has been used extensively for light input studies. However, this strain is notable as it carries the *rd1* mutation in the *Pde6b* gene (*Pde6b*^*rd1*^), which causes a rapid degeneration of photoreceptors in the retina (Pittler and Baehr, 1991). Although this mutation is useful in modeling retinal degeneration, other phenotypes (such as light responses) are likely to be confounded by the presence of *Pde6b*^*rd1*^, and so a new substrain called C3H-Pde6b+ was created to remove the *Pde6b*^*rd1*^ mutation. This was achieved by introducing the wild-type *Pde6b* allele from the BALB/c strain and backcrossing to congenic status (10 generations) (Hart et al., 2005). Although this approach successfully removed the retinal degeneration phenotype from the C3H-Pde6b+ strain, it also has introduced regions of BALB/c genome into C3H-Pde6b+. Given these genetic differences, the C3H-Pde6b+ line cannot be considered as merely the C3H-HeH line with the *Pde6b*^*rd1*^ mutation removed but as a genetically similar but distinct strain, and we therefore expect phenotypic differences between the 2 strains that cannot be explained as simply because of the *Pde6b*^*rd1*^ mutation. It is also notable that once phenotypic differences between the C3H-HeH and C3H-Pde6b+ strains have been established we can use techniques such as haplotype analysis to map regions of the BALB/c genome that are causative for the divergent phenotypes and thus identify the genes which underlie these differences. However, such studies again require baseline phenotyping comparisons of the 2 strains to identify the differences needed to undertake these more long term genetic investigations.

To address the need for more comprehensive phenotyping of these mouse strains we constructed a phenotyping pipeline that allows the study of both visual and nonvisual retinal responses, in addition to circadian rhythms and sleep in the same cohort of animals. This approach combines classic circadian wheel running activity monitoring (Banks and Nolan, 2011) and visual phenotyping assays (slit lamp and optokinetic drum) with 2 novel methods of phenotyping—assessment of the nonvisual pupillary light response (PLR) and immobility-defined sleep (Fisher et al., 2012). Using this pipeline of phenotyping techniques, we report here on the effect of aging on all these processes and furthermore show that several of these changes are strain specific.

## 2 Methods

### 2.1 Mice and test pipeline

All animal studies described in this article were performed under the guidance issued by the Medical Research Council in Responsibility in the Use of Animals for Medical Research (July 1993) and Home Office Project Licences 30/2686 and 30/3070. When not being tested, mice were housed in individually ventilated cages under 12/12 hours light/dark (LD) conditions with food and water available *ad libitum*. Four different inbred mouse strains were used: C57BL/6J, C57BL/6N, C3H-HeH (abbreviated to C3H), and C3H-Pde6b+ (abbreviated to C3PDE–see Hart et al., 2005). Female mice were used for all cohorts. Phenotyping tests were performed in the following order: pupillometry, circadian wheel running, sleep analysis by video tracking, and visual phenotyping. The animals had 1 week rest intervals between tests. Animal cohorts began testing at 5 different ages: approximately 3, 6, 9, 12, and 18 months. A separate cohort was bred and aged for each time point. 8–10 animals were used for each experiment with the following exceptions: 6 months C57BL/6J, n = 7 and 18 months C3PDE, n = 6.

### 2.2 Visual phenotyping

Visual acuity was assessed by head tracking response to a virtual-reality optokinetic system as described by Douglas et al. (2005) and manufactured by CerebralMechanics Inc (Alberta, Canada). Briefly, mice were placed onto a podium in an area comprising computer monitors as walls and a mirrored floor. The mouse was monitored by a camera built into the lid of the arena. A vertical sine wave rotates around the monitors, and the head and neck movements of the mouse are used to assess how well the mouse tracks the sine wave rotation. The spatial frequency of the lines is increased until there is no longer a response from the animal, indicating that the stimulus is no longer perceived. Grating is measured in cycles per degree. Illuminance within the apparatus was 30 lux.

The anterior segment, including lens and cornea was assessed using a slit lamp as described in Thaung et al. (2002). Briefly, mice were restrained by light scruffing and held up to the slit lamp, where the light is centered onto the mouse's eye. The procedure was performed in a dark room with the light of the slit lamp being slowly brightened to ensure pupil constriction.

### 2.3 Pupillometry

A high power neutral white LED (Luxeon star) was used to provide the illumination to generate the pupillary light reflex (light intensity of 14.4 log quanta/cm^2^/s [169 μW/cm^2^/s or 500 lux]). The pupillary response was recorded using a Prosilica near-infrared (NIR) sensitive CCD video camera (BRSL, Newbury, UK) positioned adjacent to the contralateral eye to the light source, allowing consensual light responses to be recorded. A NIR LED was used to illuminate the eye during dark periods. To take recordings, the mice were restrained in the correct position by scruffing according to normal handling techniques. Mice were dark adapted for at least 1 hour before recordings were made. All recordings were made between Zeitgeber times 4 and 8 (ZT4 and ZT8, where the onset of the light phase is defined as ZT0). The total recording time for each session was 30 seconds. A baseline measure for 2 seconds was used to establish dark adapted pupil size. A 10-second light stimulus was then applied to elicit pupillary constriction. Following this, recordings were taken for a further 18 seconds to monitor recovery in the absence of illumination. The camera was linked to a laptop via the National Instruments Measurement and Acquisition software (version 4.7.6). Data were collected using a custom made LabView application (Pothecary et al., manuscript in preparation), which also controlled the timing of both the light stimulus delivery and image recording, taking an image of the eye once every 0.2 seconds. ImageJ (http://rsbweb.nih.gov/ij/) was used to establish the area of the pupil in each image, and the magnitude of the changes in pupil size was expressed as area relative to the fully dilated pupil from the dark adapted recording.

### 2.4 Circadian activity

Circadian wheel running analysis was performed as outlined in Banks and Nolan (2011). Briefly, mice were singly housed in cages containing running wheels. Cages were placed in light-controlled chambers and wheel running activity monitored via ClockLab (Actimetrics). Animals were monitored for 7 days in a 12-hour LD cycle (100 lux light intensity). Following this, the animals were further monitored for 12 days in constant darkness (DD). Data collection and most of the analysis were performed using ClockLab. Additional analysis of the interdaily stability and intradaily variability (Van Someren et al., 1999) was performed by exporting the data to the Actiwatch activity and sleep analysis 5 software package (Cambridge Nanotechnology).

### 2.5 Video tracking of sleep behavior

Video tracking was performed as described in Fisher et al. (2012). Briefly, mice were singly housed and placed in light-controlled chambers with NIR miniature CCD cameras positioned above the cages (Maplin, UK). Monitoring during dark periods was performed using infrared illumination. Mice were allowed to acclimatize to the home cage for 24 hours in a 12-hour light/dark cycle (100 lux light intensity) before data collection. Video monitoring was then performed for a 24-hour period over a 12-hour light/dark cycle. During a second 24-hour period, mice were subjected to a 1-hour light pulse at ZT16. Mice were video monitored throughout the pulse. Video files were uploaded to ANYmaze video analysis software (Stoetling). ANYmaze was then used to track mouse mobility and to score periods during which the animals remained immobile for 40 seconds or more.

### 2.6 Statistical analysis

Two-way analysis of variance (ANOVA) was performed on data sets with “mouse strain” and “mouse age” as interaction factors. Post hoc Tukey test was also performed as part of the 2-way ANOVA. This assessed whether there was an overall difference between strains, between ages, and between how strains change with age. If aging effects were found for a specific phenotype, 1-way ANOVAs were performed for each individual strain to confirm any aging differences, with age as the interaction factor. Significance level for all statistical analysis was set at *p* < 0.05. Data are presented as mean ± standard error of the mean. For correlations between data sets a principal component analysis (PCA) was performed which included a Bartlett test of sphericity and a cut off for small coefficient values of less than 3. To confirm the interactions suggested by the PCA, Pearson correlations were performed between all the parameters within each component. All statistics were performed using SPSS (IBM).

## 3 Results

### 3.1 Visual and nonvisual retinal phenotypings

The retinal and visual pathways of the mouse cohorts were measured by slit lamp analysis, optokinetic drum head tracking, and pupillometry. Supplementary Tables 1 and 2 give the complete visual phenotyping and pupillometry data respectively, with statistical analysis showing strain and aging effects.

**Fig. 1:**
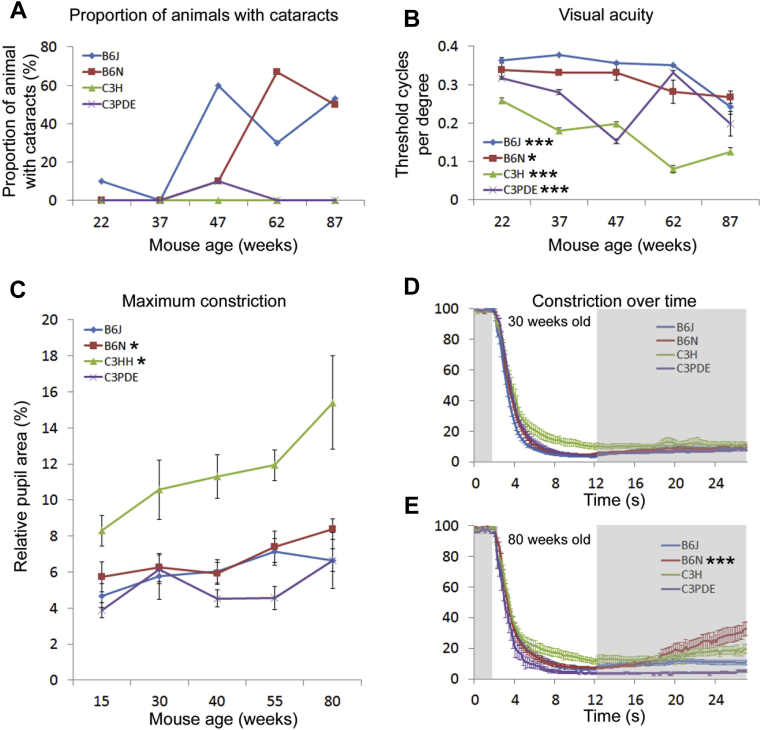
Visual and nonvisual retinal phenotypic changes with age. (A) Proportion of animals that developed cataracts with age. Note that each time point is a separate cohort of animals—the apparent reduction in the proportion of animals with cataracts at older time points does not reflect recovery but differences between cohorts (see Section 3). (B) Age-related changes in visual acuity as measured by optokinetic function in the 4 mouse strains. (C) Age-related changes in the minimum relative pupil area in dark adapted pupillometry in the 4 mouse strains. (D and E) Kinetic analysis of pupil constriction and recovery during pupillometry in 30 weeks (D) and 80 weeks (E) old animals. Stars adjacent to strain labels indicate the significance of age-related changes (* = *p* ≤ 0.05; *** = *p* ≤ 0.001). Error bars show mean ± SEM. Abbreviation: SEM, standard error of the mean.

The presence of cataracts in the eyes was assessed by observation using a slit lamp. Animals were scored by whether or not a cataract was present in one or both eyes. We found a strong age-related tendency to develop cataracts in the 2 C57BL/6 strains. It was also notable that the C57BL/6J strain started to develop cataracts at a younger age than C57BL/6N. By contrast, we only found a single cataract in our C3 entire cohort and found no age-related prevalence for cataracts in these strains (Fig. 1A). We note that, in our data the proportion of cataracts in some of our strains declines in older animals. This does not reflect recovery of cataracts in individual animals. This is because each age group is a separate cohort of animals, and the changes in cataract prevalence reflect differences between these cohorts. We could find no obvious relationship between housing conditions and the incidence of cataracts (data not shown).

The visual acuity of our animal cohorts was assessed by head-tracking measurements on the optokinetic drum. Two-way ANOVA analysis of our optokinetic drum data demonstrated that there were significant differences in visual acuity between strains (F[3,162] = 133.857; *p* ≤ 0.00001), ages (F[4,162] = 32.485; *p* ≤ 0.00001), and in the interaction between age and strain (F[12,162] = 13.236; *p* ≤ 0.00001). Further, analysis showed that mice of all strains showed an age-related reduction in acuity as indicated by a decrease in the spatial frequency at the threshold of head tracking (1-way ANOVA for optokinetic drum threshold; *p* < 0.05 for all strains) (Fig. 1B). Notably, the acuity of the 2 C57BL/6 strains showed no significant differences when compared with each other (2-way ANOVA, interaction factors “age × strain”; F[12,162] = 13.236; post hoc Tukey *p* = 0.102 for comparisons between C57BL/6J and C57BL/6N), but by contrast, the C3 strains had significantly lower acuity than the C57BL/6 strains (2-way ANOVA, interaction factors “age × strain”; F[12,162] = 0.997; post hoc Tukey *p* < 0.05 for comparisons between C57BL/6 and C3 strains), and the acuity for the C3H strain was significantly lower than for C3PDE (2-way ANOVA, interaction factors “age × strain”; F[12,162] = 0.997; post hoc Tukey *p* < 0.05 for comparisons between C3H and CPDE strains).

The physiological response to light was assessed by the pupillary light response (PLR) of our animal cohorts, using a white light source (Fig. 1C). A 2-way ANOVA analysis of our data demonstrated that there were significant differences in maximum constriction between mouse strains (F[3,162] = 36.98; *p* ≤ 0.00001) and ages (F[4,162] = 5.9; *p* = 0.0002) but not in the age and strain interaction (F[12,162] = 0.997; *p* = 0.454). One-way ANOVAs conducted on individual strains showed that aging has no effect on maximum pupil constriction in the C57BL/6J and C3PDE strains, but the maximum pupil constriction of the C3H and C57BL/6N strains was significantly reduced with age (1-way ANOVA for maximum constriction; *p* < 0.05 for C57BL/6N and C3H strains). We also performed a more in-depth analysis of the PLR by tracking pupil size over time to show how age may affect the kinetics and recovery of the response (Fig. 1D and E). Previous studies have demonstrated that the amount of functional photoreceptors in the retina will alter the maximum relative constriction of the PLR but not the rate of constriction (Hughes et al., 2012). Therefore, to assess whether the neuronal networks underlying the PLR undergo an age-related decline such as that seen in some other brain regions (Palomba et al., 2008); we analyzed the rate of pupil constriction in our animal cohorts. Following, the light stimulus the pupil size initially decreases rapidly before the speed of constriction slows in the later stages of the response. We therefore divided the constriction phase of the PLR into early (the first second following light stimulation, characterized by rapid pupillary constriction) and late (the final 9 seconds of light stimulation, characterized by slower or no constriction) stages for analysis purposes. We found no age-related differences in the rate of constriction during the early stage of the PLR. However, during the late stage of the PLR, the speed of constriction was significantly slower in older animals (between 55 and 80 weeks) in the 2 C57BL/6 strains (1-way ANOVA for rate of constriction; *p* < 0.05 for C57BL/6 strains). The late stage constriction rate was unaffected by age in the 2 C3 strains.

Following the cession of the light pulse, we also analyzed the rate at which the pupil recovers. A 2-way ANOVA analysis of our data demonstrated that there were significant differences in post illumination recovery between mouse strains (F[3,162] = 5.612; *p* = 0.001), ages (F[4,162] = 9.159; *p* = 0.000002), and the age and strain interaction (F[12,162] = 7.204; *p* ≤ 0.00001). Further, analysis using 1-way ANOVAs demonstrated that there was no effect of age on the speed of post illumination recovery in the C57BL/6J or either C3 strains. However in the C57BL/6N strain, older (80 weeks old) animals showed a significant increase in the rate of pupil recovery following the termination of the light stimulus (1-way ANOVA for rate of recovery; *p* < 0.05 for C57BL/6N) (Fig. 1E). Before this age the recovery rate for the C57BL/6N strain showed no age-related changes.

In addition to the age-related changes in PLR reported previously, we also noted that at all ages the maximum pupil constriction of the C3H strain was significantly reduced compared with that of other strains (2-way ANOVA, interaction factors “age × strain”; F[12,162] = 0.997; post hoc Tukey *p* < 0.05 for comparisons between C3H and all other strains).

### 3.2 Circadian activity

**Fig. 2:**
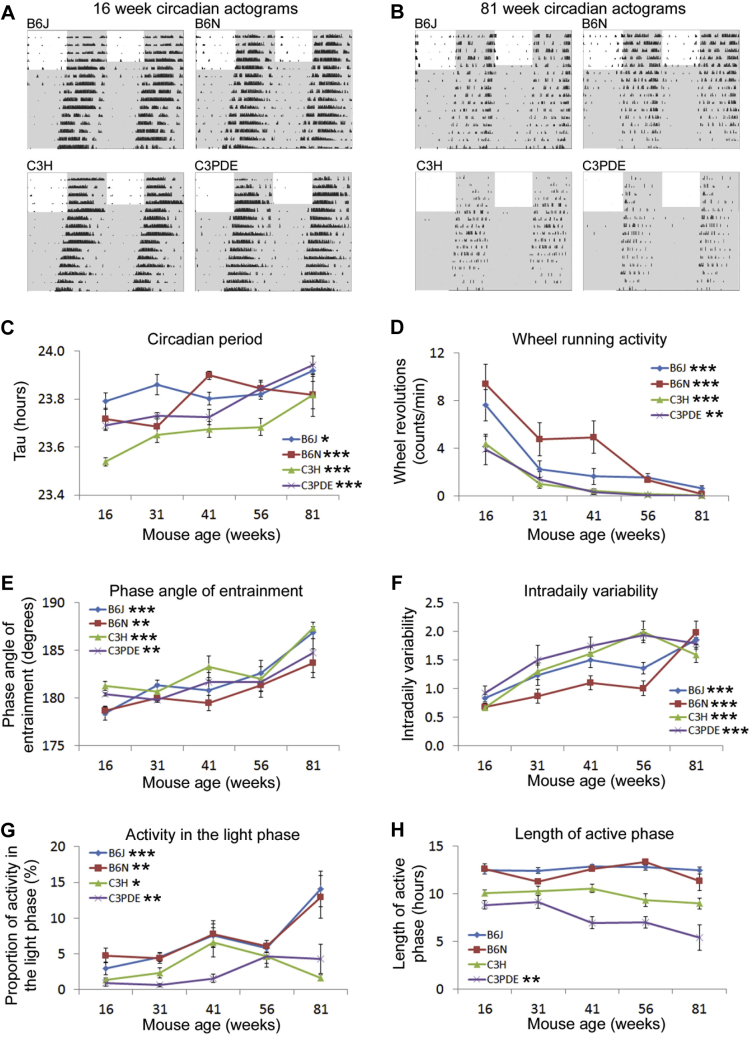
Circadian analysis of inbred strains across age. (A and B) Representative double plotted actograms from 16 weeks (A) and 81 weeks (B) old animals. Shaded regions represent periods where the animals are in darkness. Vertical black bars represent wheel running activity. See Banks and Nolan (2011) for a full explanation of double plotted actograms. (C) Age-related changes in the circadian period (τ) in the 4 mouse strains. (D) Age-related changes in wheel running activity in the 4 mouse strains in constant darkness. (E) Age-related changes in the phase angle of entrainment in the 4 mouse strains. (F) Age-related changes in intradaily variability in the 4 mouse strains. (G) Age-related changes in the proportion of wheel running activity in the light phase of the cycle in the 4 mouse strains. (H) Age-related changes in the length of active phase (alpha) in the 4 mouse strains in constant darkness. Stars adjacent to strain labels indicate the significance of age-related changes (* = *p* ≤ 0.05; ** = *p* ≤ 0.01; *** = *p* ≤ 0.001). Error bars show mean ± SEM. Abbreviation: SEM, standard error of the mean.

Wheel running analysis was performed for 7 days in a 12-hour light/dark (LD) cycle. Following this, wheel running was monitored for a further 12 days in constant darkness (DD). Representative actograms are shown for 16-week-old mice (Fig. 2A) and 81-week-old mice (Fig. 2B). Supplementary Tables 3 and 4 show the complete circadian data obtained and statistical analysis to characterize the effect of aging.

Analysis of our data by 2-way ANOVA tests demonstrated that all circadian parameters differed significantly between strain, age, and in the interaction between strain and age (Supplementary Tables 3 and 4). Further analysis was therefore performed to characterize these differences. In all strains, circadian period (τ) significantly lengthened with age (1-way ANOVA for each strain; *p* < 0.05) (Fig. 2C). This lengthening was most pronounced in the 2 C3 strains analyzed (between 16 and 81 weeks C3H lengthened by 17 ± 4 minutes and C3PDE lengthened by 15 ± 3 minutes). The lengthening in the 2 C57 strains was milder but still significant (between 16 and 81 weeks C57BL/6J lengthened by 8 ± 3 minutes and C57BL/6N lengthened by 6 ± 6 minutes). Cumulative wheel running activity, in both LD and DD conditions, significantly decreased with age in all mouse strains (1-way ANOVA for each strain; *p* < 0.05), with the most rapid decline occurring during early aging (Fig. 2D). This is most pronounced in DD conditions where activity between the ages of 16 and 31 weeks dropped by 70%, 49%, 76%, and 63% for C57BL/6J, C57BL/6N, C3H, and C3PDE, respectively. The phase angle of entrainment became progressively more delayed with age in all strains indicating that in relation to light cycles, mice delay their onset of their activity as they age (1-way ANOVA for each strain; *p* < 0.05) (Fig. 2E). We also noted, from observing the actograms obtained in our studies, that as mice of all strains age their wheel running rhythms became fragmented. This is reflected in a progressive loss of circadian amplitude and an increase in intradaily variability with age (1-way ANOVA for each strain; *p* < 0.05) (Fig. 2F). Although we observed no clear strain differences in the phase angle of entrainment or amplitude we did note that the intradaily variability of 31- to 56-week-old C57BL/6N animals in constant darkness was significantly lower than that of other strains (2-way ANOVA, interaction factors “age × strain”; F[12,162] = 2.95; post hoc Tukey *p* < 0.05 for C57BL/6N compared with all other strains). The same trend was observed for the intradaily variability of C57BL/6N animals in light-dark cycles, but it was not significant in this condition. In contrast to intradaily variability, interdaily stability in LD showed no significant age-related changes.

The phenotypes described previously showed the same age-related trends in all mouse strains. However, we also identified strain specific differences in the effect of aging on the proportion of wheel running activity in the light (inactive) phase of LD and the length of the active phase (α) of the animals. The proportion of wheel running activity in the light phase of LD significantly increased with age for the C57BL/6 and C3PDE strains (1-way ANOVA for each strain; *p* < 0.05). For the C57BL/6 strains, this increase was greatest during later life (between 56 to 81 weeks the C57BL/6J and C57BL/6N strains increased by 8.1% and 6.8%, respectively, whereas between 16 and 56 weeks the C57BL/6J and C57BL/6N strains increased by 2.8% and 1.3%, respectively). For the C3PDE strain, the increase was greatest between 41 and 56 weeks (increase of 3.1%) with mild to no increase between 16 and 41 weeks (0.6% increase) and a very mild decrease from 56 and 81 weeks (0.4% decrease). In contrast, the C3H strain showed an increase in the activity in the light phase between the ages of 16 and 41 weeks, after which the activity decreased so that by 81 weeks of age the activity was equal to that of 16-week-old animals (1-way ANOVA; *p* < 0.05) (Fig. 2G). The length of the animal's active phase (α) also showed strain-specific aging differences. α for the C57BL/6 and C3H strains in LD and DD conditions remained consistent with mouse age, whereas C3PDE animals showed a mild age-related shortening of α in both conditions (1-way ANOVA for C3PDE; *p* < 0.05 in both LD and DD) (Fig. 2H).

In addition to the age-related changes described previously, we also noted some aging independent phenotypic differences between strains. When comparing the 2 C57BL/6 strains with the 2 C3 strains, we noted that for all ages C57BL/6 strains had an increased proportion of activity in the light phase (2-way ANOVA, interaction factors “age × strain”; F[12,162] = 3.309; post hoc Tukey *p* < 0.05 for comparisons between the C57BL/6 and C3 strains), increased wheel running activity in both LD and DD conditions (2-way ANOVA, interaction factors “age × strain”; F[12,162] = 1.664; post hoc Tukey *p* < 0.05 for comparisons between the C57BL/6 and C3 strains) and a longer α (2-way ANOVA, interaction factors “age × strain”; F[12,162] = 3.067; post hoc Tukey *p* < 0.05 for comparisons between the C57BL/6 and C3 strains) compared with the 2 C3 strains. Additionally, we noted some differences that were unique to specific substrains. C3H animals had a shorter τ than all other strains (2-way ANOVA, interaction factors “age × strain”; F[12,162] = 4.069; post hoc Tukey *p* < 0.05 for C3H compared with all other strains). C3PDE animals had a shorter α than all other strains (2-way ANOVA, interaction factors “age × strain”; F[12,162] = 3.067; post hoc Tukey *p* < 0.05 for C3PDE compared with all other strains). C57BL6/N animals had a higher wheel running activity in both LD and DD conditions than all other strains (2-way ANOVA, interaction factors “age × strain”; F[12,162] = 1.664; post hoc Tukey *p* < 0.05 for C57BL/6N compared with all other strains). Finally, C57BL/6N animals had a higher circadian amplitude than either of the C3 strains (2-way ANOVA, interaction factors “age × strain”; F[12,162] = 2.553; post hoc Tukey *p* < 0.05 for comparisons between C57BL/6N and C3 strains).

### 3.3 Immobility-defined sleep

Immobility-defined sleep was assessed both over a 24-hour period (in a 12-hour light/dark cycle) and during/following a 1-hour light pulse given during the dark phase (ZT16-17) of a second 24-hour period. Animals were video recorded throughout the test periods given previously, and the videos analyzed using ANYmaze video tracking software. This allowed bouts of immobility lasting 40 seconds or more (corresponding to sleep) to be scored (Pack et al., 2007). Additionally, animal activity levels (both distance traveled and velocity) were measured by the video tracking software. Supplementary Tables 5 and 6 show the complete video tracking data obtained and statistical analysis to characterize the effect of aging.

**Fig. 3:**
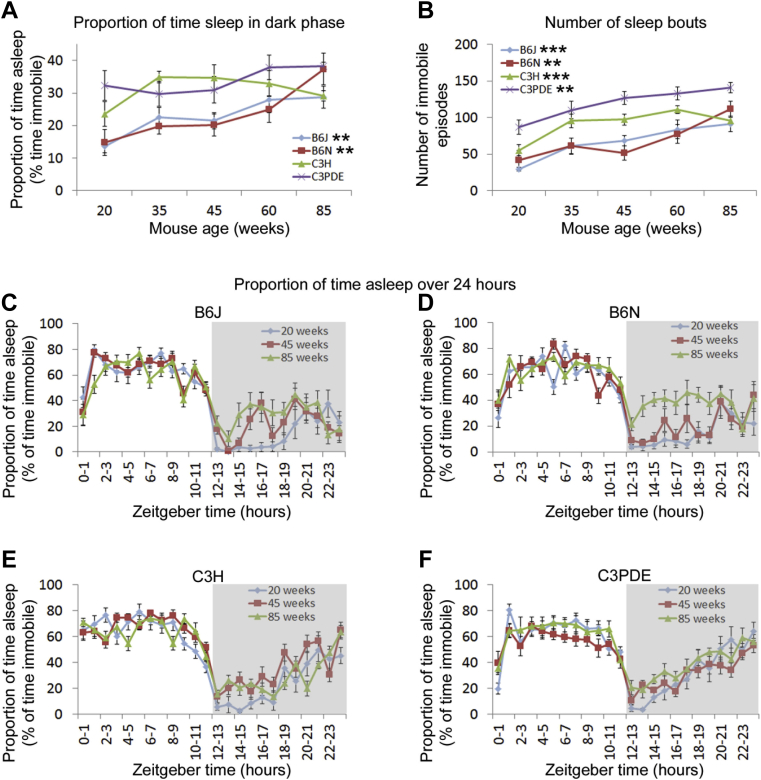
Video-tracking analysis of sleep changes with age. (A) Age-related changes in the proportion of time spent asleep in the 4 mouse strains in the dark phase. (B) Age-related changes in the number of sleep bouts in the 4 mouse strains in the dark phase. (C–F) Age-related changes in sleep and/or wake behavior over 24 hours in C57BL/6J (C), C57BL/6N (D), C3H (E), and C3PDE (F) strains. For clarity, data are only shown for 20, 45, and 85 weeks as representative time points throughout the life span of the mouse. Stars adjacent to strain labels indicate the significance of age-related changes (** = *p* ≤ 0.01; *** = *p* ≤ 0.001). Error bars show mean ± SEM. Abbreviation: SEM, standard error of the mean.

Data analysis by 2-way ANOVA tests demonstrated that all sleep parameters measured differed significantly between mouse strains and ages, although we found no differences in the interaction between strain and age (Supplementary Tables 3 and 4). Further analysis was therefore performed to characterize these differences. We found that for both C57BL/6 strains, the proportion of time spent asleep over 24 hours increased with age (1-way ANOVA for proportion of time immobile; *p* < 0.05 for C57BL/6 strains). Further analysis demonstrated that this increase was because of an increase in sleep amount in the dark phase of the 24 hour period (ZT 12–24; night time) (1-way ANOVA for proportion of time immobile in active phase; *p* < 0.05 for C57BL/6 strains) (Fig. 3A). Sleep amount in the light phase (ZT 0–12; day time) remained constant with age. In contrast, neither of the 2 C3 mouse strains showed significant age-related differences in the proportion of time spent asleep, either in total or in the active or inactive phases of the cycle.

Although the previously mentioned data analyze the total sleep amount over 24 hours, we also analyzed the patterns of sleep throughout the 24 hour period. This was achieved by separating the sleep measurements into 1 hour time bins across the analyzed day. In the light phase, sleep was maintained at a constant level in all strains and ages. However, in the dark phase we observed 3 distinct patterns of sleep. C57BL/6J mice showed a biphasic sleep pattern in the dark phase, with 2 peaks in sleep amount at approximately ZT 15–16 (early night) and ZT 20–22 (late night). This pattern showed variations with age with young (20 weeks) mice showing no early night peak, and old (85 weeks) mice showing no distinct trough in sleep between the 2 peaks (Fig. 3C). C57BL/6N mice showed no clear pattern of sleep throughout the dark phase. Although this did not change with age, the proportion of time asleep increased in older animals (Fig. 3D). C3 strains show a gradual increase in the amount of sleep as they progress through the dark phase. This pattern did not appear to change with age (Fig. 3E and F).

In immobility-defined sleep measurements, each shift from an immobile to mobile state is a measure of a transition from sleep to waking. Therefore, immobility-defined sleep can be used to assess sleep fragmentation by measuring the number of immobility bouts over 24 hours—an increase in immobility bouts reflects an increase in sleep fragmentation as the animal wakes more often. We found that sleep fragmentation increased with age in all mouse strains (1-way ANOVA for total number of immobility bouts; *p* < 0.05 for all strains). Further analysis demonstrated that this increase in sleep fragmentation was because of increased fragmentation in the dark phase (1-way ANOVA for number of immobility bouts in active phase; *p* < 0.05 for all strains) (Fig. 3B). Sleep fragmentation in the light phase remained constant with age.

Consistent with the circadian wheel running data shown previously, the activity parameters measured by video tracking (total distance moved and average velocity) both showed an age-related decrease in all strains (1-way ANOVAs distance moved in video tracking and average velocity for each strain; *p* < 0.05). Again the most rapid decline in activity occurred during early life with total distance traveled dropping by 41%, 42%, 72%, and 36% for C57BL/6J, C57BL/6N, C3H, and C3PDE respectively between the ages of 20 and 35 weeks.

As with the circadian data described previously, we also noted some age-independent differences between the mouse strains. At all ages, C57BL/6 strains displayed higher activity measures (both total distance and average velocity) than C3 strains (2-way ANOVA, interaction factors “age × strain”; post hoc Tukey *p* < 0.05 for comparisons between the C57BL/6 and C3 strains). Additionally, C57BL/6 strains had fewer sleep episodes in the dark phase than the C3 strains (2-way ANOVA, interaction factors “age × strain”; F[12,149] = 1.261; post hoc Tukey *p* < 0.05 for comparisons between the C57BL/6 and C3 strains)—that is, the C57BL/6 strains showed less fragmentation of sleep than C3 strains. Furthermore, we noted that C3H mice had fewer sleep bouts than C3PDE animals, suggesting that C3PDE had more fragmented sleep than C3H (2-way ANOVA, interaction factors “age × strain”; F[12,149] = 1.261; post hoc Tukey *p* < 0.05 for comparisons between the C3H and C3PDE strains).

**Fig. 4:**
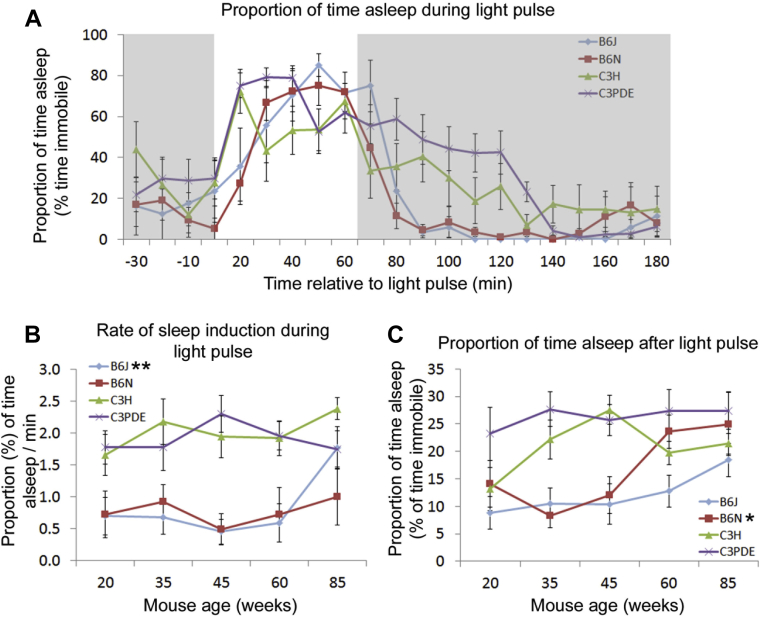
Changes in the sleep response to light with age. (A) Age-related changes in the proportion of time spent asleep in the 4 mouse strains during and following a light pulse in the dark phase. Representative data are shown for 35-week-old animals. Shaded regions represent time in darkness in the protocol. (B) Age-related changes in the rate of induction of sleep in the first 20 minutes of the light pulse in the 4 mouse strains. (C) Age-related changes in the total proportion of time spent asleep in the 2 hours following the end of the light pulse. Stars adjacent to strain labels indicate the significance of age-related changes (* = *p* ≤ 0.05; ** = *p* ≤ 0.01). Error bars show mean ± SEM. Abbreviation: SEM, standard error of the mean.

In mice, sleep can be induced by subjecting animals to a light pulse in the dark phase. To assess how aging affects this response in different strains, mice were given a 1-hour light pulse at ZT16. Video-tracking analysis was performed for 30 minutes before the pulse, for the duration of the pulse and for 2 hours subsequent to the pulse. Analysis was performed on data separated into 10 minutes time bins. From this analysis, we observed that the light pulse induced a period of immobility-defined sleep in mice of all strains and ages (Fig. 4A). Two-way ANOVA tests were used to analyze the proportion of time asleep both during and after the light pulse and also the speed at which the animals fell asleep during the pulse and the speed at which they awoke following the pulse (Supplementary Table 6). We found no significant effect of age or strain on the total proportion of time spent asleep during the light pulse. We also found no effect of age on the speed of sleep induction by the light pulse or the offset of sleep (waking) after the light pulse (Fig. 4B and Supplementary Table 6). However, we did find significant differences between mouse strains in the speed of sleep induction, the proportion of time spent asleep after the light pulse, and the offset of sleep after the light pulse. Additionally, we found a significant difference in the age and strain interaction in the proportion of time spent asleep following the light pulse. We therefore analyzed these effects further. Using 1-way ANOVA, we found no significant age-related differences in the total time asleep in the 2 hours following the light pulse in the C57BL/6J and C3PDE strains. However, there was a significant age-related increase in the total time asleep in the 2 hours following the light pulse in the C57BL/6N and C3H strains (1-way ANOVA for total proportion of time asleep in the 2 hours following the LP; *p* < 0.05 for C57BL/6N and C3H strains) (Fig. 4C).

We also noted some age-independent differences between strains in respect to their response to the light pulse. Sleep onset in response to the light pulse was significantly faster in C3 strains than in C57BL/6 strains (2-way ANOVA on the proportion of time immobile per minute for the first 20 minutes of the light pulse, interaction factors “age × strain”; F[12,149] = 0.621; post hoc Tukey *p* < 0.05 for comparisons between C3 strains and C57BL/6 strains) (Fig. 4B). This difference in sleep onset was further highlighted by the time taken to achieve a peak in time asleep following the start of the light pulse. C3 mouse strains reached a peak in the proportion of time asleep between 10 and 30 minutes after the onset of the light pulse whereas B6 strains reacted slower, reaching a peak in time asleep between 30 and 50 minutes after the light pulse. Additionally, we found that the C3 strains spent significantly more time asleep in the 2 hours following the light pulse than the C57BL/6 strains (2-way ANOVA on proportion of time asleep in the 2 hours following the light pulse, interaction factors “age × strain”; F[12,149] = 1.626; post hoc Tukey *p* < 0.05 for comparisons between C3 strains and C57BL/6 strains).

### 3.4 Phenotypic correlations

Because this study generated a large data set describing a range of phenotypic parameters in the same strains, we decided to establish whether there were any strong correlations among the phenotypes described here. This was initially carried out using principal component analysis (PCA). PCA was performed on data using all parameters described above and in the Supplementary Tables. Data were taken from all animals regardless of strain or age to ascertain possible correlations independent of these factors.

The PCA gave rise to a 10-component solution accounting for 81.7% of total variance (Supplementary Table 7). To confirm the correlations suggested by the PCA, we then performed Pearson correlations between all the parameters within each component. In most of the cases the Pearson correlations between the parameters of each component were significant (*p* < 0.05) confirming the PCA groupings. However, we did note the following exceptions. In component 1, interdaily stability in LD was not significantly correlated with circadian amplitude in LD, the number of sleep episodes in the dark, or the intradaily variability in both LD and DD (*p* > 0.1). Additionally, we noted that the PCA loading score for interdaily stability in LD was low compared with other parameters in this component, and the loading score for interdaily stability in LD for component 1 was very close to that of component 3 (0.459 for component 1 and 0.458 for component 3). We therefore concluded that interdaily stability in LD was only weakly associated with component 1. In component 5, circadian period was not significantly associated with the other parameters of the component (Pearson correlations of −0.24, *p* = 0.168 for both correlations between “proportion of time asleep in the dark phase of LD” and “circadian period” and between “total proportion of time asleep in LD” and “circadian period”). We therefore decided to exclude circadian period from this component. In component 7, the total sleep episodes during LD was not significantly correlated with the proportion of time spent asleep in the light (Pearson correlation: −0.06, *p* = 0.441). In component 8, the 2 parameters making up the component (proportion of circadian activity in the light and maximum relative constriction during PLR) were not significantly correlated (Pearson correlation: −0.16, *p* = 0.831). We therefore discarded component 8. Additionally components 9 and 10, both accounted for a single parameter (“speed of pupil recovery following light induced constriction” and “speed of waking following light pulse” for components 9 and 10, respectively), suggesting that these 2 parameters did not correlate with any others. We therefore also discarded components 9 and 10.

Adjusted PCA analysis gives rise to a 7-component solution (Table 1). Component 1 contained a number of activity measures and circadian measures heavily influenced by activity. We therefore loosely interpreted component 1 as “general activity and activity-influenced circadian measures.” Component 2 contained a number of activity measures in the light phase of LD and circadian measures in LD that are set by the onset of the light phase. We therefore loosely interpreted component 2 as “light influenced general activity and circadian measures.” Likewise, component 3 was interpreted as “sleep measures following a light pulse,” component 4 as “pupillary constriction rates,” component 5 as “proportion of time sleeping,” component 6 as “sleep during a light pulse,” and component 7 as “sleep during the light phase.” The measures of circadian period, the speed of pupillary recovery following PLR, the maximum relative constriction during PLR, and the speed of waking following a light pulse were not included in components with other parameters.

**Table 1:** PCA of phenotype parameters followed by Pearson correlation analysis within components. The variance accounted by each component is shown in the table in brackets below the component number

	Notes from Pearson correlations	Component						
		1	2	3	4	5	6	7
		(25.2%)	(12%)	(7.3%)	(6.5%)	(6.1%)	(5.9%)	(5.2%)
Circadian activity in DD		**0.912**						
Circadian activity in dark phase of LD		**0.911**						
Total circadian activity in LD		**0.908**						
Distance traveled in dark by video tracking		**0.885**						
Speed of movement in dark by video tracking		**0.885**						
Total distance traveled by video tracking		**0.879**						
Speed of movement in light and dark by video tracking		**0.879**						
Circadian amplitude in LD		**0.752**						
Circadian amplitude in DD		**0.741**						
Number of sleep episodes in dark		**−0.633**		0.458		0.419		
Intradaily variability in DD		**−0.542**		0.390				
Intradaily variability in LD		**−0.513**		0.303				
Interdaily stability in LD	No correlations with amplitude in LD, number of sleep episodes in dark and intradaily variability	**0.459**		0.458				
Length of active phase in LD			**0.778**					
Phase angle of entrainment			**−0.726**					
Speed of movement in light by video tracking		0.326	**0.704**					
Length of active phase in DD			**0.693**					
Distance traveled in light by video tracking		0.326	**0.693**				−0.319	
Optokinetic drum score		0.319	**0.618**					
Circadian activity in light phase of LD		0.409	**0.566**					
Number of sleep episodes following light pulse				**0.824**				
Proportion of time asleep following light pulse				**0.783**				
Initial stage PLR constriction rate					**0.936**			
Late stage PLR constriction rate					**−0.931**			
Proportion of time asleep in the dark phase	Circadian period removed from component 5	−0.498				**0.749**		
Total proportion of time asleep		−0.448				**0.691**		−0.318
Number of sleep episodes during light pulse							**0.808**	
Proportion of time asleep during light pulse							**0.708**	
Speed of sleep induction during light pulse			−0.381				**0.504**	
Number of sleep episodes during light phase								**0.881**
Total number of sleep episodes		−0.549		0.371				**0.558**
Proportion of time asleep in the light phase	Not correlated with total sleep episodes in light						0.547	**−0.552**

Values in bold highlight the highest PCA loading scores for that parameter and therefore show how parameters are clustered into specific components.

Key: DD, constant darkness; LD, light:dark; PCA, principal component analysis.

## 4 Discussion

A detailed discussion of our results is given below. Table 2 highlights the key age and strain-related phenotypes we have identified. Also included in Table 2 are some candidate genes that may contribute to some of the phenotypes found (see Section 4 in the following).

**Table 2:** Summary of key findings

Phenotype	Age-related changes		Non aging strain differences	Possible genes of interest
	Aging effect	In strain		
Constriction during PLR	Decreased	C57BL/6N; C3H	Poorer in C3H	*Guca1a* (Kamenarova et al., 2013)
	Maintained	C57BL/6J; C3PDE		*Crb1* (Mattapallil et al., 2012)
				*Pde6b* (Hart et al., 2005)
Optokinetic head tracking	Decreased	All	Poorer in C3H	*Pde6b* (Hart et al., 2005) *Crb1* (Simon et al., 2013)
Appearance of cataracts	Increased	C57BL/6		
	None	C3		
Tau	Lengthened	All	Shorter in C3H	
Proportion of activity in light	Increased	C57BL/6; C3PDE		
	Increased up to 41 wk, decrease after	C3H		
Amplitude	Decreased	All		
Intra daily variability	Decreased	All		
Alpha	Maintained	C57BL/6; C3H (in DD)	Longer in C57BL/6	
	Shortened	C3H (in LD); C3PDE		
Phase angle of entrainment	Delayed	All		
Activity	Decreased	All	Higher in C57BL/6N	*Adcy5* (Simon et al., 2013)
				*Pmch* (Simon et al., 2013)
Proportion of sleep in dark phase	Increased	C57BL/6	Higher in C3	*Aanat* (Roseboom et al., 1998) *Asmt* (Kasahara et al., 2010)
	Maintained	C3		
Sleep bouts	Increased	All	Higher in C3PDE	
Speed of sleep induction during light pulse	Maintained	All	Faster in C3	

Key: DD, constant darkness; LD, light:dark; PLR, pupillary light response.

Advances in modern medicine and an increased focus on healthy lifestyles have greatly increased life expectancies in recent times. This has, in turn, led to an increasing interest in healthy aging, broadly defined as “the process of optimizing opportunities for health, participation, and security to enhance quality of life as people age” (World Health Organization, 2002). As a result, understanding the effect of aging on processes such as sleeping or light responsiveness has never been more relevant. One of the most powerful tools we have in aiding this understanding are animal models, the most common of which is the laboratory mouse. The data we have presented here demonstrate the effect of age in 4 different mouse strains on visual and nonvisual retinal responses, circadian rhythms, and sleep and/or wake timing, providing a complete phenotypic data set of these interacting pathways.

Human studies have demonstrated that during aging there is a decline in visual acuity (Matheson et al., 1998) and an increase in the prevalence of cataracts (Klein and Klein, 2013). Our data demonstrate that C57BL/6 strains provide a good model of these changes, as both strains studied show a reduction in optokinetic drum assayed visual acuity and an increase in the incidence of cataracts with age. Interestingly, although the C3 strains both show an age-related reduction in optokinetic drums scores, we found no increase in the prevalence of cataracts with age in these strains. Previous studies have demonstrated that an F1 hybrid of C57BL/6NIAA and C3H/NIAA animals develop cataracts later in life than pure strain C57BL/6NIAA animals (Wolf et al., 2000). Coupled with the data presented here, this strongly suggests that the C3 mouse strains carry genetic factors that suppress cataract development. In addition to visual acuity and cataract data we have also characterized the PLR. Pupillometry studies in humans have demonstrated that aging has no effect on light-induced pupil constriction (Daneault et al., 2012). We also found no evidence of an age-related decline in PLR in 2 of our mouse strains (C57BL/6J and C3PDE). It is notable that, as described previously, the C57BL/6J strain shows an age-related increase in cataracts but no age-related decline in PLR, suggesting that the presence of cataracts alone is not enough to disrupt the pupillary response. These findings are consistent with the fact that the PLR primarily responds to environmental irradiance rather than the spatial information that is required for image-forming responses to light. Overall, with regards the visual and nonvisual phenotyping presented here, we note that the C57BL/6J strain is the closest to a human model of aging in that it shows a decline in visual acuity, a prevalence for cataract formation and no change in PLR with age.

A number of candidate genes have been identified that may underlie these strain differences in visual and nonvisual phenotyping. The most striking of these is the *Pde6b* gene, which encodes the β-subunit of rod-specific cyclic-GMP phosphodiesterase. The C3H strains carry a mutation in this gene, known as *rd1* (*Pde6b*^*rd1*^), that causes a rapid loss of rod photoreceptors and a more protracted loss of cone photoreceptors (Carter-Dawson et al., 1978, Pittler and Baehr, 1991). The C3PDE strain was generated to remove the *Pde6b*^*rd1*^ mutation from the C3 background by introducing the wild-type *Pde6b* allele from the BALB/c strain and backcrossing to congenic status (Hart et al., 2005). Using this approach, the C3H and C3PDE strains will genetically differ not only in the presence of the *Pde6b*^*rd1*^ allele, but also because of spontaneous mutations that have become fixed by genetic isolation and regions of the BALB/c genome will still be present from the founding backcrosses (although subsequent backcrosses to C3H will have progressively removed most of the BALB/c genome some may still remain, particularly in regions in proximity to the *Pde6b* gene, which have been selected for retention to maintain the wild-type allele). However, in the case of the visual and nonvisual phenotyping presented here, we are confident that the differences between C3H and C3PDE in visual acuity and PLR can be ascribed as being because of the *Pde6b*^*rd1*^ mutation. Previous studies have demonstrated that mice lacking rods and cones are less sensitive to the light stimulus required to produce a full PLR response (Lucas et al., 2001), and that animals which lack rod and cone function show reduced optokinetic drum scores (Schmucker et al., 2005). Such studies demonstrate that a lack of functional rods and cones (as occurs in *Pde6b*^*rd1*^-induced retinal degeneration) can account for the visual and nonvisual phenotypic differences described here. We also note that previous studies have found animals carrying the *Pde6b*^*rd1*^ allele are unable to head track in visual acuity tests (Hart et al., 2005). However, it has been demonstrated that mice lacking functional rods are able to resolve gratings at high light intensities because of surviving cones (Schmucker et al., 2005). The light intensity used in these studies is comparable with the intensity in our optokinetic apparatus. Given this and the protracted loss of cones in *Pde6b*^*rd1*^ induced degeneration, the head-tracking response in our C3H animals may be attributed to the surviving population of cones in the retina, and the age-related decline in visual acuity in this strain reflects the gradual loss of cones in the eye.

A recent article by Simon et al. (2013) has reported on ophthalmological differences between the C57BL/6J and C57BL/6N strains. In contrast to our data, Simon et al. (2013) found a significant reduction in visual acuity measured by optokinetic drum in C57BL/6N compared with C57BL/6J. Although we found no overall difference between the 2 C57BL/6 strains in acuity, we do note that for all but the oldest animals the drum scores of the C57BL/6N animals were lower than for C57BL/6J. With regards to the PLR data presented here, we also note that while the C57BL/6N strain did show an age-related decline in their PLR, the actual effect of this was mild—at no specific age did the maximum constriction of the PLR significantly differ between the C57BL/6J and C57BL/6N strains. Overall, therefore, the visual and retinal defects of the C57BL/6N strain are mild and possibly not biologically relevant. C57BL/6N mice carry a point mutation in the *Crb1* gene—a gene known to be causative for *rd8* retinal degeneration (Mattapallil et al., 2012). The degeneration associated with *rd8* is characterized by the appearance of folds in the retinal layers (known as pseudorosettes) and may be caused by impaired adhesion between retinal cells (Aleman et al., 2011, van de Pavert et al., 2007). *Crb1* is therefore a strong candidate to be causative for the visual and/or retinal differences that we show here. Moreover, Simon et al. (2013) report a coding difference between the 2 strains in the gene *Guca1a*. *Guca1a* encodes a guanylate cyclase activating protein, mutations in which have been associated with loss of photoreceptors (Kamenarova et al., 2013). Because both the *Crb1* and *Guca1a* genes are strong candidates for ophthalmic phenotypes, further studies are required to ascertain the underlying genetic cause for the phenotypes described here and elsewhere.

In our circadian wheel running analysis and consistent with previous studies using C57BL/6J animals (Farajnia et al., 2012, Possidente et al., 1995, Valentinuzzi et al., 1997), we observed an age-related decrease in amplitude, an increase in intradaily variability, a decrease in total average daily activity, an increase in activity in the light phase of the cycle, and an advance in the phase angle of entrainment in all 4 mouse strains analyzed here. In addition to these, we also found a shortening of the active phase (α) of older C3 mice which was not observed in C57BL/6 animals. Although previous studies have demonstrated that C57BL/6J mice do show a shorter α in old age, this reduction was not observed until the mice were between 500 and 700 days old (Farajnia et al., 2012). Previous reports have also shown that the τ of C57BL/6J mice lengthen with age (Farajnia et al., 2012, Possidente et al., 1995, Valentinuzzi et al., 1997). Our data here confirm this and further demonstrate that the same age-related increase in τ in C57BL/6J is observed in C57BL/6N and C3 strains.

The sleep data presented here were obtained through the use of immobility-defined sleep analysis of video-tracked animals. Although the benefits of video-tracking analysis of sleep have been discussed elsewhere (Fisher et al., 2012, Pack et al., 2007), it is notable that both the video-tracking data presented here and previous studies using classical EEG sleep measurements show comparable age-related phenotypes in C57BL/6J mice (e.g., increase in sleep in dark phase and increased sleep fragmentation with age) (Eleftheriou et al., 1975, Hasan et al., 2012). The replication of these previous findings clearly demonstrates the validity of video tracking as a method of sleep analysis.

Our analysis noted a strain-specific aging phenotype in the proportion of time spent asleep in the dark phase of the cycle. We found that in the dark phase C3 strains maintained a constant amount of sleep with age although C57BL/6 strains increased their amount of sleep as they got older. Notably, all strains increased the number of sleep bouts in the dark phase as they aged. In the case of aged C57BL/6 animals, the increase in the proportion of time asleep and increase in number of sleep bouts in the dark phase simply implies that the animals show a greater propensity to sleep throughout the dark phase of the cycle. However, aged C3 animals spend the same proportion of time asleep in the dark phase as young animals, and yet show an increase in the number of sleep bouts, suggesting that their dark phase sleep becomes more fragmented with age. In humans, a number of age-related sleep phenotypes have been reported, including fragmentation of sleep and an increase in daytime napping (Huang et al., 2002). The C57BL/6 and C3 strains therefore appear to model 2 different aspects of human aging sleep phenotypes: C57BL/6 animals show an increase in the proportion of time spent asleep in the dark phase (the equivalent of day time napping), whereas the C3 animals show increased sleep fragmentation with age.

When considering circadian rhythms and sleep, one of the most cited genetic differences between C57BL/6 and C3 mouse strains is in melatonin synthesis. Early studies noted that C57BL/6J animals have no detectable melatonin in their pineal glands (Ebihara et al., 1986). This was later demonstrated to be because of C57BL/6J animals expressing a truncated form of the enzyme arylalkylamine N-acetyltransferase and severely reduced expression of the enzyme hydroxyindole O-methyltransfease (Kasahara et al., 2010, Roseboom et al., 1998), which are required for melatonin production. A more recent study by Simon et al. (2013), which compared the C57BL/6J and C57BL/6N genomes did not identify differences between these genes in the 2 genomes, suggesting that the arylalkylamine N-acetyltransferase and hydroxyindole O-methyltransfease defects are also present in the C57BL/6N strain. These genes have been demonstrated to be expressed normally in C3H animals, and given the lineage of the C3PDE strain, it is highly likely that these genes are also correctly expressed in C3PDE animals. It has been suggested that the reduction in melatonin expression which occurs during aging is responsible for age-related changes seen in sleep (Grad and Rozencwaig, 1993). However, our data do not support this, as we observed only mild differences between the melatonin deficient and melatonin proficient strains. Our data therefore support others who have suggested that melatonin has only a mild effect on sleep regulation (Fisher and Sugden, 2010).

We have also used immobility-defined sleep to characterize the sleep response to a light pulse applied in the dark phase of the cycle. Human studies into the effect of aging on light responsiveness have used light pulses to induce phase shifting responses in melatonin, body temperature, and activity cycles and compared these phase shifting responses between age groups. Such studies have shown that the light responsiveness of humans is either not affected (Kim et al., 2014, Kripke et al., 2007) or only mildly altered (Klerman et al., 2001) by aging. Although light pulse induction of sleep, such as described here, is not directly comparable with all the human data stated previously, the fact that we observed no aging effect on sleep induction by light pulses further demonstrates the effectiveness of the mouse as a model organism of that aging process. We also note that we observed no C3H specific effect on the sleep induction (although the C3H strain reacted faster to the light pulse than C57BL/6 strains, there was no difference between the C3H and C3PDE strains). Therefore, the induction of sleep was unaffected by the presence of the *Pde6b*^*rd1*^ mutation described previously. This is likely because of the fact that the photosensitive retinal ganglion cells which can drive sleep responses to light (Lupi et al., 2008) have been demonstrated to be resistant to *Pde6b*^*rd1*^ induced degeneration (Lin and Peng, 2013).

Although we have discussed previously some of the known genes that may influence the phenotypic differences reported here, more in-depth genomic analyses and comparisons across the different strains will highlight other possible candidate genes (see Table 2). Two recent studies have performed these kinds of analyses comparing the phenotypes and genomes of C57BL/6J and C57BL/6N strains using either next generation sequencing to identify all the genomic differences between the strains (Simon et al., 2013) or using QTL analysis to link a specific phenotype to a specific mutation (Kumar et al., 2013). These studies have identified a number of gene differences between the strains, some of which may influence the circadian or sleep phenotypes described here. These genes include *Nkapl* and *Pde4b* (both of which have been associated with schizophrenia; Kirsty Millar et al., 2005, Yue et al., 2011), *Adcy5* (modulates dopaminergic transmission and can influence behavior and activity levels (Kim et al., 2008), *Pmch* (reported to act as a neurotransmitter and/or neuromodulator in goal directed behavior and arousal (Mul et al., 2011), and *Cyfip2* (plays a role in establishing neuronal connectivity (Pittman et al., 2010). Further comparative studies of the genomes of the strains reported here will identify additional candidate genes for the strain and aging differences highlighted here. However, the genetic differences between the C3H and C3PDE strains should be noted. As previously stated, the C3PDE strain was created to remove the *Pde6b*^*rd1*^allele from the C3H background. However, this does not mean that all of the phenotypic differences between these 2 strains will be because of the *Pde6b*^*rd1*^allele (see above). For example, although we found a difference in τ and α between C3H and C3PDE, the presence of *Pde6b*^*rd1*^ in the C57BL/6J mouse strain has previously been shown to produce no differences in circadian behavior (Foster et al., 1991). It is therefore likely that strain differences such as these are because of the presence of remnants of the BALB/c genome in the C3PDE strain that remain from the founding outcross used in the strain's creation (Hart et al., 2005).

Overall, there is clear evidence that aging has a disruptive effect on circadian rhythms, sleep, and light responsive pathways (for examples see Dijk et al., 1999, Huang et al., 2002). The data presented here demonstrate that although the effects of aging in different mouse strains are broadly the same in terms of how visual and nonvisual retinal responses, circadian rhythms, and sleep are altered, the genetic heterogeneity of the different strains leads to subtle phenotypic variations in the aging effect. Understanding this phenotypic variance is of great importance not only when using different mouse strains as a tool in aging studies but also in the study of how genetic differences can affect the aging process in individuals. Some of the genetic differences between the mouse strains have been characterized and are available (Simon et al., 2013) and the advances in low cost whole-genome sequencing means that the remaining strain differences can easily be identified. Comparing such genetic techniques and analyses with phenotyping data sets such as those presented here will help to identify candidate genes and networks that can modulate and potentially alleviate the disruptive effects of aging, and thus provide a basis for improvements in the quality of life for the elderly individuals.

## Disclosure statement

The authors declare no conflicts of interest in this study.
