# Supplementary material for: Genetic background influences age-related decline in visual and nonvisual retinal responses, circadian rhythms, and sleep
Source: Neurobiol Aging. 2015 Jan;36(1):380–93. doi: 10.1016/j.neurobiolaging.2014.07.040 (PMC4270439; doi:10.1016/j.neurobiolaging.2014.07.040)
Supplement: Supplementary Tables 1 – 7 [file mmc1.docx]

Supplementary data.

Table S1: Visual phenotyping.

|  | | | **Parameter** | |
| --- | --- | --- | --- | --- |
|  |  |  | **Optokinetic drum score (threshold cycles / degree)** | **Proportion of animals with cataracts (%)** |
| **B6J** | **Age (weeks)** | **22** | 0.363 ±0.008 | 10 |
|  |  | **37** | 0.377 ±0.002 | 0 |
|  |  | **47** | 0.355 ±0.003 | 60 |
|  |  | **62** | 0.35 ±0.002 | 30 |
|  |  | **87** | 0.242 ±0.02 | 53 |
|  | **1 way ANOVA (age)** | **F (4,41)** | 19.378 | NA |
|  |  | **P** | ≤0.0000001 | NA |
| **B6N** | **Age (weeks)** | **22** | 0.339 ±0.021 | 0 |
|  |  | **37** | 0.331 ±0.007 | 0 |
|  |  | **47** | 0.312 ±0.021 | 10 |
|  |  | **62** | 0.281 ±0.03 | 67 |
|  |  | **87** | 0.267 ±0.015 | 50 |
|  | **1 way ANOVA (age)** | **F (4,39)** | 3.158 | NA |
|  |  | **P** | 0.022 | NA |
| **C3H** | **Age (weeks)** | **22** | 0.258 ±0.007 | 0 |
|  |  | **37** | 0.179 ±0.008 | 0 |
|  |  | **47** | 0.199 ±0.005 | 0 |
|  |  | **62** | 0.08 ±0.01 | 0 |
|  |  | **87** | 0.125 ±0.009 | 0 |
|  | **1 way ANOVA (age)** | **F (4,43)** | 75.241 | NA |
|  |  | **P** | ≤0.0000001 | NA |
| **C3PDE** | **Age (weeks)** | **22** | 0.317 ±0.003 | 0 |
|  |  | **37** | 0.281 ±0.007 | 0 |
|  |  | **47** | 0.153 ±0.006 | 10 |
|  |  | **62** | 0.332 ±0.004 | 0 |
|  |  | **87** | 0.198 ±0.032 | 0 |
|  | **1 way ANOVA (age)** | **F (4,38)** | 73.741 | NA |
|  |  | **P** | ≤0.0000001 | NA |
| **2-way ANOVA** | **Age** | **F (4,162)** | 32.485 | NA |
|  |  | **P** | ≤0.0000001 | NA |
|  | **Strain** | **F (3,162)** | 133.857 | NA |
|  |  | **P** | ≤0.0000001 | NA |
|  | **Age X Strain** | **F (12,162)** | 13.236 | NA |
|  |  | **P** | ≤0.0000001 | NA |

Table S2: Pupillometry data.

|  | | | **Parameter** | | | |
| --- | --- | --- | --- | --- | --- | --- |
|  |  |  | **Maximum constriction (Relative pupil area %)** | **Initial constriction rate (Relative pupil area change/sec)** | **Late stage constriction rate (Relative pupil area change/sec)** | **Post illumunation recovery rate (Relative pupil area change/sec)** |
| **B6J** | **Age (weeks)** | **15** | 4.68 ±0.66 | -54.5 ±2.37 | -3.7 ±0.21 | 0.11 ±0.05 |
|  |  | **30** | 5.76 ±1.27 | -45.7 ±3.63 | -4.7 ±0.41 | 0.29 ±0.08 |
|  |  | **40** | 6.04 ±0.65 | -40.2 ±4 | -5.31 ±0.48 | 0.12 ±0.05 |
|  |  | **55** | 7.12 ±0.74 | -43.3 ±3.5 | -4.85 ±0.34 | 0.19 ±0.1 |
|  |  | **80** | 6.66 ±0.62 | -50.1 ±10.1 | -4.93 ±0.39 | 0.17 ±0.06 |
|  | **1 way ANOVA (age)** | **F (4,41)** | 1.56 | 1.26 | 2.75 | 1.04 |
|  |  | **P** | 0.2024 | 0.308 | 0.048 | 0.406 |
| **B6N** | **Age (weeks)** | **15** | 5.72 ±0.83 | -48.3 ±3.16 | -4.49 ±0.39 | 0.06 ±0.05 |
|  |  | **30** | 6.25 ±0.78 | -37 ±2.14 | -5.56 ±0.31 | 0.32 ±0.09 |
|  |  | **40** | 5.93 ±0.6 | -37.1 ±1.4 | -5.54 ±0.23 | 0.07 ±0.05 |
|  |  | **55** | 7.4 ±0.87 | -31.5 ±3 | -6.3 ±0.35 | 0.13 ±0.05 |
|  |  | **80** | 8.39 ±0.57 | -50 ±10.5 | -4.78 ±0.4 | 1.69 ±0.28 |
|  | **1 way ANOVA (age)** | **F (4,39)** | 2.62 | 1.92 | 4.14 | 22.17 |
|  |  | **P** | 0.0494 | 0.145 | 0.014 | ≤0.0000001 |
| **C3H** | **Age (weeks)** | **15** | 8.3 ±0.85 | -40.1 ±2.43 | -4.88 ±0.2 | 0.19 ±0.11 |
|  |  | **30** | 10.58 ±1.65 | -32.5 ±3.04 | -5.44 ±0.51 | 0.13 ±0.05 |
|  |  | **40** | 11.31 ±1.21 | -34.9 ±2.97 | -5.38 ±0.23 | 0.38 ±0.17 |
|  |  | **55** | 11.93 ±0.84 | -31 ±4.21 | -5.55 ±0.39 | 0.18 ±0.14 |
|  |  | **80** | 15.4 ±2.59 | -37.8 ±4.22 | -4.92 ±0.44 | 0.47 ±0.28 |
|  | **1 way ANOVA (age)** | **F (4,43)** | 2.61 | 1.15 | 0.84 | 0.84 |
|  |  | **P** | 0.0489 | 0.349 | 0.511 | 0.513 |
| **C3PDE** | **Age (weeks)** | **15** | 3.87 ±0.43 | -42.6 ±3.24 | -4.89 ±0.33 | 0.11 ±0.12 |
|  |  | **30** | 6.15 ±0.78 | -30.7 ±2.82 | -6.16 ±0.31 | 0.23 ±0.07 |
|  |  | **40** | 4.54 ±0.48 | -40 ±4.98 | -5.24 ±0.58 | 0.22 ±0.06 |
|  |  | **55** | 4.56 ±0.65 | -32.3 ±3.66 | -6.16 ±0.42 | 0.04 ±0.04 |
|  |  | **80** | 6.65 ±1.56 | -47.1 ±4.74 | -4.33 ±0.71 | 0.11 ±0.08 |
|  | **1 way ANOVA (age)** | **F (4,38)** | 2.45 | 2.74 | 2.63 | 1.4 |
|  |  | **P** | 0.0623 | 0.067 | 0.054 | 0.256 |
| **2-way ANOVA** | **Age** | **F (4,162)** | 5.9 | 5.661 | 6.226 | 9.159 |
|  |  | **P** | 0.00019 | 0.00035 | 0.00014 | 0.000002 |
|  | **Strain** | **F (3,162)** | 36.98 | 6.009 | 3.001 | 5.612 |
|  |  | **P** | 0 | 0.00078 | 0.034 | 0.00129 |
|  | **Age X Strain** | **F (12,162)** | 0.997 | 0.538 | 1.061 | 7.204 |
|  |  | **P** | 0.454 | 0.886 | 0.4 | ≤0.0000001 |

Table S3: Circadian data in light dark cycles (LD).

|  | | | **Parameter** | | | | | | |
| --- | --- | --- | --- | --- | --- | --- | --- | --- | --- |
|  |  |  | **Activity (wheel rotations per min)** | **Proportion of activity in light phase (%)** | **Phase angle of entrainment** | **Amplitude** | **Alpha (hours)** | **Interdaily Stability** | **Intradaily Variability** |
| **B6J** | **Age (weeks)** | **16** | 4.4 ±0.92 | 2.98 ±0.87 | 178.4 ±0.7 | 690.5 ±38 | 11.59 ±0.09 | 0.67 ±0.05 | 0.69 ±0.08 |
|  |  | **31** | 1.4 ±0.36 | 4.57 ±0.63 | 181.3 ±0.56 | 597.1 ±79 | 11.71 ±0.16 | 0.55 ±0.07 | 1.29 ±0.14 |
|  |  | **41** | 1.6 ±0.79 | 7.59 ±1.58 | 180.8 ±1.08 | 593.1 ±59 | 12.05 ±0.2 | 0.6 ±0.05 | 1.42 ±0.13 |
|  |  | **56** | 1.1 ±0.22 | 5.84 ±0.68 | 182.6 ±1.35 | 569.5 ±50 | 11.75 ±0.12 | 0.54 ±0.04 | 1.28 ±0.12 |
|  |  | **81** | 0.6 ±0.17 | 14.07 ±2.45 | 186.9 ±0.63 | 509.3 ±21 | 12.03 ±0.19 | 0.5 ±0.01 | 1.71 ±0.09 |
|  | **1 way ANOVA (age)** | **F (4,40)** | 6.04 | 8.52 | 8.7 | 1.84 | 1.8 | 2.09 | 11.56 |
|  |  | **P** | 0.0007 | 0.00005 | 0.00004 | 0.1399 | 0.1475 | 0.101 | 0.000003 |
| **B6N** | **Age (weeks)** | **16** | 5.3 ±1.03 | 4.77 ±1.04 | 178.7 ±0.47 | 716.5 ±47 | 11.87 ±0.22 | 0.71 ±0.08 | 0.61 ±0.06 |
|  |  | **31** | 2.9 ±0.97 | 4.41 ±0.71 | 180 ±0.33 | 634.5 ±27 | 11.59 ±0.24 | 0.67 ±0.06 | 0.78 ±0.08 |
|  |  | **41** | 4.8 ±1.3 | 7.78 ±1.89 | 179.5 ±0.78 | 759.8 ±66 | 12.01 ±0.14 | 0.66 ±0.07 | 0.72 ±0.09 |
|  |  | **56** | 1.3 ±0.25 | 6.08 ±0.81 | 181.3 ±1.21 | 542 ±31 | 12.07 ±0.2 | 0.59 ±0.05 | 1.31 ±0.15 |
|  |  | **81** | 0.3 ±0.18 | 12.95 ±2.95 | 183.7 ±1.49 | 466.2 ±30 | 11.68 ±0.43 | 0.57 ±0.04 | 2.38 ±0.36 |
|  | **1 way ANOVA (age)** | **F (4,40)** | 5.14 | 4.26 | 4.19 | 6.62 | 0.77 | 0.89 | 19.22 |
|  |  | **P** | 0.0019 | 0.0057 | 0.0064 | 0.00035 | 0.5531 | 0.48 | ≤0.000001 |
| **C3H** | **Age (weeks)** | **16** | 2.5 ±0.63 | 1.41 ±0.22 | 181.3 ±0.47 | 711.6 ±52 | 10.4 ±0.19 | 0.59 ±0.04 | 0.55 ±0.13 |
|  |  | **31** | 0.5 ±0.14 | 2.34 ±0.7 | 180.7 ±0.79 | 539.2 ±44 | 9.37 ±0.73 | 0.47 ±0.04 | 1.04 ±0.12 |
|  |  | **41** | 0.3 ±0.09 | 6.63 ±2.05 | 183.3 ±1.12 | 505.3 ±40 | 8.56 ±0.7 | 0.48 ±0.05 | 1.47 ±0.15 |
|  |  | **56** | 0.2 ±0.15 | 4.65 ±1.51 | 182 ±1.26 | 498.1 ±42 | 8.44 ±0.6 | 0.65 ±0.04 | 1.81 ±0.18 |
|  |  | **81** | 0.05 ±0.01 | 1.63 ±0.47 | 187.3 ±0.56 | 422.1 ±10 | 8.69 ±0.29 | 0.6 ±0.1 | 1.7 ±0.22 |
|  | **1 way ANOVA (age)** | **F (4,42)** | 10.8 | 3.39 | 7.94 | 6.6 | 2.14 | 1.99 | 10.94 |
|  |  | **P** | 0.000004 | 0.017 | 0.00007 | 0.00033 | 0.0925 | 0.113 | 0.000007 |
| **C3PDE** | **Age (weeks)** | **16** | 1.9 ±0.9 | 0.96 ±0.5 | 180.4 ±0.16 | 622.9 ±38 | 9.26 ±0.42 | 0.72 ±0.1 | 0.96 ±0.2 |
|  |  | **31** | 0.7 ±0.27 | 0.66 ±0.22 | 179.8 ±0.25 | 586.2 ±41 | 9.06 ±0.74 | 0.58 ±0.04 | 1.27 ±0.24 |
|  |  | **41** | 0.2 ±0.09 | 1.56 ±0.59 | 181.7 ±0.99 | 461.8 ±24 | 7.25 ±0.83 | 0.78 ±0.13 | 1.68 ±0.2 |
|  |  | **56** | 0.06 ±0.02 | 4.7 ±1.01 | 181.7 ±0.99 | 462.1 ±30 | 8.84 ±0.52 | 0.71 ±0.06 | 1.89 ±0.13 |
|  |  | **81** | 0.01 ±0.007 | 4.31 ±2.04 | 184.8 ±2.06 | 393 ±30 | 5.14 ±0.81 | 0.97 ±0.29 | 3.51 ±1.08 |
|  | **1 way ANOVA (age)** | **F (4,40)** | 2.94 | 5.48 | 5.22 | 6.88 | 3.53 | 1.22 | 6.37 |
|  |  | **P** | 0.032 | 0.0013 | 0.0018 | 0.0003 | 0.0155 | 0.317 | 0.0005 |
| **2-way ANOVA** | **Age** | **F (4,162)** | 16.512 | 11.41 | 1.162 | 15.37 | 3.957 | 1.13 | 31.386 |
|  |  | **P** | ≤0.0000001 | ≤0.0000001 | 0.33 | ≤0.0000001 | 0.004 | 0.344 | ≤0.000001 |
|  | **Strain** | **F (3,162)** | 14.487 | 17.216 | 12.479 | 7.282 | 78.028 | 6.785 | 11.16 |
|  |  | **P** | ≤0.0000001 | ≤0.0000001 | ≤0.0000001 | 0.0001 | ≤0.0000001 | 0.0002 | 0.000001 |
|  | **Age X Strain** | **F (12,162)** | 1.731 | 3.309 | 7.936 | 1.578 | 2.847 | 1.388 | 2.959 |
|  |  | **P** | 0.065 | ≤0.0000001 | ≤0.0000001 | 0.103 | 0.001 | 0.176 | 0.001 |

Table S4: Circadian data in constant darkness (DD).

|  | | | **Parameter** | | | | |
| --- | --- | --- | --- | --- | --- | --- | --- |
|  |  |  | **Tau (hours)** | **Activity (wheel rotations per min)** | **Amplitude** | **Alpha (hours)** | **Intradaily Variability** |
| **B6J** | **Age (weeks)** | **16** | 23.79 ±0.034 | 7.6 ±1.30 | 1397 ±62 | 12.51 ±0.13 | 0.84 ±0.06 |
|  |  | **31** | 23.86 ±0.042 | 2.3 ±0.72 | 914.7 ±140 | 12.44 ±0.3 | 1.24 ±0.18 |
|  |  | **41** | 23.80 ±0.026 | 1.7 ±0.68 | 746.7 ±54 | 12.9 ±0.2 | 1.5 ±0.13 |
|  |  | **56** | 23.82 ±0.022 | 1.6 ±0.35 | 863.7 ±108 | 12.82 ±0.31 | 1.35 ±0.1 |
|  |  | **81** | 23.92 ±0.025 | 0.69 ±0.22 | 627.2 ±43 | 12.45 ±0.34 | 1.86 ±0.09 |
|  | **1 way ANOVA (age)** | **F (4,40)** | 3.24 | 13.61 | 14.15 | 0.7 | 12.14 |
|  |  | **P** | 0.022 | ≤0.0000001 | ≤0.0000001 | 0.5934 | 0.000001 |
| **B6N** | **Age (weeks)** | **16** | 23.71 ±0.045 | 9.4 ±1.67 | 1526.7 ±113 | 12.59 ±0.53 | 0.68 ±0.07 |
|  |  | **31** | 23.68 ±0.041 | 4.8 ±1.40 | 1372.4 ±106 | 11.26 ±0.25 | 0.87 ±0.12 |
|  |  | **41** | 23.89 ±0.017 | 4.9 ±1.36 | 1002.6 ±65 | 12.6 ±0.22 | 1.1 ±0.12 |
|  |  | **56** | 23.84 ±0.034 | 1.4 ±0.22 | 722.9 ±85 | 13.35 ±0.34 | 1.01 ±0.13 |
|  |  | **81** | 23.82 ±0.089 | 0.17 ±0.10 | 508.4 ±66 | 11.38 ±1.05 | 1.99 ±0.2 |
|  | **1 way ANOVA (age)** | **F (4,40)** | 7.23 | 7.97 | 20 | 2.41 | 13.99 |
|  |  | **P** | 0.0002 | 0.00008 | ≤0.0000001 | 0.0671 | 0.0000003 |
| **C3H** | **Age (weeks)** | **16** | 23.54 ±0.017 | 4.4 ±0.66 | 1537.4 ±112 | 10.06 ±0.34 | 0.67 ±0.05 |
|  |  | **31** | 23.65 ±0.031 | 1 ±0.36 | 772.1 ±111 | 10.29 ±0.5 | 1.3 ±0.14 |
|  |  | **41** | 23.67 ±0.033 | 0.4 ±0.16 | 637.2 ±62 | 10.55 ±0.44 | 1.61 ±0.11 |
|  |  | **56** | 23.68 ±0.036 | 0.2 ±0.08 | 486.8 ±32 | 9.33 ±0.67 | 1.99 ±0.19 |
|  |  | **81** | 23.82 ±0.057 | 0.07 ±0.03 | 526.6 ±50 | 8.98 ±0.55 | 1.6 ±0.13 |
|  | **1 way ANOVA (age)** | **F (4,42)** | 7.67 | 25.31 | 26.94 | 1.65 | 14.39 |
|  |  | **P** | 0.0001 | ≤0.0000001 | ≤0.0000001 | 0.171 | 0.0000002 |
| **C3PDE** | **Age (weeks)** | **16** | 23.69 ±0.017 | 3.9 ±1.28 | 1137.9 ±124 | 8.82 ±0.45 | 0.93 ±0.12 |
|  |  | **31** | 23.73±0.013 | 1.4 ±0.60 | 834.5 ±126 | 9.13 ±0.67 | 1.51 ±0.25 |
|  |  | **41** | 23.73 ±0.033 | 0.3 ±0.18 | 582.7 ±52 | 6.95 ±0.63 | 1.75 ±0.16 |
|  |  | **56** | 23.85 ±0.025 | 0.07 ±0.02 | 506.1 ±33 | 7 ±0.61 | 1.94 ±0.09 |
|  |  | **81** | 23.94 ±0.038 | 0.02 ±0.009 | 429.3 ±19 | 5.39 ±1.36 | 1.79 ±0.1 |
|  | **1 way ANOVA (age)** | **F (4,40)** | 13.54 | 5.48 | 9.34 | 4.24 | 6.05 |
|  |  | **P** | ≤0.0000001 | 0.0013 | 0.00002 | 0.00606 | 0.001 |
| **2-way ANOVA** | **Age** | **F (4,162)** | 12.626 | 33.38 | 61.755 | 4.035 | 33.35 |
|  |  | **P** | ≤0.0000001 | ≤0.0000001 | ≤0.0000001 | 0.004 | ≤0.0000001 |
|  | **Strain** | **F (3,162)** | 15.861 | 13.814 | 12.45 | 106.818 | 9.402 |
|  |  | **P** | ≤0.0000001 | ≤0.0000001 | ≤0.0000001 | ≤0.000001 | 0.00001 |
|  | **Age X Strain** | **F (12,162)** | 4.069 | 1.664 | 2.553 | 3.067 | 2.95 |
|  |  | **P** | 0.00002 | 0.079 | 0.004 | 0.001 | 0.001 |

Table S5: Video tracking data over 24 hours.

|  | | | **Parameter** | | | | | | | |
| --- | --- | --- | --- | --- | --- | --- | --- | --- | --- | --- |
|  |  |  | **Total proportion of time asleep (% time immobile)** | **Proportion of sleep in light (% time immobile)** | **Proportion of sleep in dark (% time immobile)** | **Total sleep episodes (no of immobile episodes)** | **Sleep episodes in light (no of immobile episodes)** | **Sleep episodes in dark (no of immobile episodes)** | **Distance moved (m)** | **Velocity (m/s)** |
| **B6J** | **Age (weeks)** | **16** | 38.4 ±0.92 | 63.2 ±1.2 | 13.6 ±2.1 | 189.1 ±5.2 | 159.5 ±5.9 | 29.6 ±2.5 | 1384.8 ±176 | 0.016 ±0.002 |
|  |  | **31** | 45.1 ±1.87 | 67.8 ±0.9 | 22.5 ±3.1 | 215.7 ±12.5 | 154.7 ±3 | 61 ±11.1 | 810.5 ±140 | 0.009 ±0.002 |
|  |  | **41** | 41.8 ±1.03 | 62.2 ±1.4 | 21.5 ±2.5 | 235.7 ±12.6 | 167.2 ±7.3 | 68.5 ±7.1 | 493.5 ±66 | 0.006 ±0.001 |
|  |  | **56** | 46.8 ±2.33 | 65.7 ±1.9 | 27.9 ±4 | 237.5 ±14.8 | 153.8 ±7.1 | 83.8 ±12.7 | 338.5 ±64 | 0.004 ±0.001 |
|  |  | **81** | 44.1 ±2.16 | 59.5 ±1.6 | 28.7 ±3.4 | 255 ±13.2 | 163.6 ±3.9 | 91.4 ±10.9 | 224.6 ±47 | 0.003 ±0.001 |
|  | **1 way anova (age)** | **F (4,37)** | 4.14 | 4.07 | 4.44 | 4.91 | 0.85 | 8 | 17.91 | 17.86 |
|  |  | **P** | 0.007 | 0.078 | 0.005 | 0.003 | 0.5043 | 0.00009 | ≤0.00001 | ≤0.0001 |
| **B6N** | **Age (weeks)** | **16** | 37.24 ±2.06 | 59.7 ±1.2 | 14.8 ±4.2 | 196.8 ±15.2 | 154.8 ±7.2 | 42 ±13.2 | 1647.8 ±434 | 0.019 ±0.005 |
|  |  | **31** | 42.29 ±1.35 | 64.7 ±1.2 | 19.8 ±2.5 | 230.2 ±10.2 | 169 ±8.3 | 61.2 ±10.4 | 959.7 ±220 | 0.011 ±0.003 |
|  |  | **41** | 40.67 ±2.19 | 61.3 ±1.7 | 20.1 ±3.4 | 199.5 ±14.8 | 147.9 ±10.8 | 51.6 ±10 | 1244.4 ±379 | 0.015 ±0.004 |
|  |  | **56** | 42.94 ±2.6 | 60.9 ±3.1 | 25 ±4 | 238.4 ±14.2 | 160.9 ±9.3 | 77.5 ±12.8 | 429.2 ±106 | 0.005 ±0.001 |
|  |  | **81** | 50.13 ±4.08 | 62.9 ±3.6 | 37.3 ±4.9 | 270.8 ±12 | 159.2 ±7.5 | 111.6 ±11 | 150.8 ±55 | 0.002 ±0.001 |
|  | **1 way anova (age)** | **F (4,41)** | 3.4 | 0.74 | 5.01 | 5.16 | 0.81 | 5.62 | 4.08 | 4.09 |
|  |  | **P** | 0.017 | 0.5697 | 0.0022 | 0.002 | 0.5246 | 0.0011 | 0.0071 | 0.007 |
| **C3H** | **Age (weeks)** | **16** | 43.98 ±2.32 | 64.4 ±2.9 | 23.5 ±3.8 | 204.1 ±10.3 | 148.8 ±4.9 | 55.3 ±7.4 | 764.7 ±110 | 0.009 ±0.001 |
|  |  | **31** | 50.61 ±1.31 | 66.4 ±2 | 34.9 ±1.8 | 254.4 ±10.7 | 159 ±3.6 | 95.4 ±9.1 | 210.6 ±53 | 0.002 ±0.001 |
|  |  | **41** | 50.92 ±1.75 | 67.1 ±1.8 | 34.8 ±3.9 | 254.9 ±9.7 | 157.6 ±4.1 | 97.3 ±7.8 | 143.8 ±30 | 0.002 ±0.0004 |
|  |  | **56** | 48.48 ±2.16 | 64.1 ±1.1 | 32.8 ±4 | 275.5 ±6.3 | 164.6 ±3.3 | 110.9 ±5.1 | 158.9 ±29 | 0.002 ±0.0003 |
|  |  | **81** | 46.67 ±1.25 | 64.3 ±2.1 | 29.1 ±1.6 | 270.3 ±10.4 | 174.6 ±7.4 | 95.6 ±6.1 | 146.9 ±25 | 0.002 ±0.0002 |
|  | **1 way anova (age)** | **F (4,37)** | 2.55 | 0.47 | 2.06 | 8.15 | 3.7 | 7.79 | 21.72 | 21.82 |
|  |  | **P** | 0.055 | 0.7564 | 0.1062 | 0.00008 | 0.0125 | 0.0001 | ≤0.00001 | ≤0.0001 |
| **C3PDE** | **Age (weeks)** | **16** | 46.53 ±3.03 | 60.8 ±2.5 | 32.3 ±4.6 | 236.3 ±11.3 | 149.1 ±8.3 | 87.1 ±9.6 | 383.2 ±101 | 0.004 ±0.001 |
|  |  | **31** | 46.25 ±2.54 | 62.8 ±1.7 | 29.7 ±3.9 | 273.2 ±10.9 | 163.1 ±5.7 | 110.1 ±11.9 | 244.2 ±51 | 0.003 ±0.001 |
|  |  | **41** | 43.53 ±2.27 | 56.1 ±3.5 | 30.9 ±4.1 | 296 ±9.7 | 169.3 ±7.4 | 126.8 ±8.9 | 129.6 ±16 | 0.001 ±0.0002 |
|  |  | **56** | 50.03 ±2.93 | 62.2 ±2.3 | 37.9 ±3.8 | 301.8 ±13.5 | 168.4 ±6.4 | 133.4 ±8.8 | 91.1 ±11 | 0.001 ±0.0001 |
|  |  | **81** | 50.27 ±1.89 | 62.2 ±2.7 | 38.3 ±1.2 | 312.2 ±9.3 | 171.6 ±10.2 | 140.6 ±7.1 | 84.4 ±9 | 0.001 ±0.0001 |
|  | **1 way anova (age)** | **F (4,34)** | 1.01 | 1.17 | 0.94 | 6.49 | 1.45 | 4.08 | 5.06 | 5.21 |
|  |  | **P** | 0.414 | 0.3413 | 0.4505 | 0.0005 | 0.2386 | 0.0083 | 0.0026 | 0.0022 |
| **2-way ANOVA** | **Age** | **F (4,149)** | 4.935 | 1.978 | 6.635 | 19.507 | 2.001 | 19.266 | 14.833 | 14.702 |
|  |  | **P** | 0.0009 | 0.101 | 0.00006 | ≤0.0000001 | 0.097 | ≤0.00001 | ≤0.00001 | ≤0.0001 |
|  | **Strain** | **F (3,149)** | 7.654 | 4.031 | 11.264 | 24.188 | 0.609 | 29.604 | 16.386 | 16.446 |
|  |  | **P** | 0.00008 | 0.0086 | 0.000001 | ≤0.0000001 | 0.61 | ≤0.00001 | ≤0.00001 | ≤0.0001 |
|  | **Age X Strain** | **F (12,149)** | 1.643 | 1.006 | 1.633 | 1.176 | 1.129 | 1.261 | 1.99 | 2.019 |
|  |  | **P** | 0.086 | 0.447 | 0.088 | 0.305 | 0.341 | 0.248 | 0.029 | 0.026 |

Table S6: Video tracking data over during light pulse.

|  | | | **Parameter** | | | |
| --- | --- | --- | --- | --- | --- | --- |
|  |  |  | **Proportion of time asleep during light pulse (% time immobile)** | **Speed of sleep induction by light pulse (% time immobile/min)** | **Proportion of time asleep following light pulse (% time immobile)** | **Speed of waking following light pulse (% time immobile/min)** |
| **B6J** | **Age (weeks)** | **16** | 49.71 ±6.55 | 0.7 ±0.29 | 8.85±2.97 | -1.42 ±0.62 |
|  |  | **31** | 56.99 ±7.12 | 0.68 ±0.27 | 10.54 ±2.78 | -3.57 ±0.61 |
|  |  | **41** | 49.74 ±2.49 | 0.45 ±0.19 | 10.41 ±3.69 | -1.53 ±0.57 |
|  |  | **56** | 57.14 ±5.75 | 0.59 ±0.3 | 12.76 ±2.91 | -1.75 ±0.55 |
|  |  | **81** | 58.81 ±3.83 | 1.77 ±0.33 | 18.43 ±3.06 | -1.81 ±0.82 |
|  | **1 way ANOVA (age)** | **F (4,37)** | 0.73 | 2.7 | 1.34 | 1.52 |
|  |  | **P** | 0.5746 | 0.053 | 0.2725 | 0.216 |
| **B6N** | **Age (weeks)** | **16** | 52.29 ±3.24 | 0.73 ±0.36 | 14.08 ±4.25 | 0.14 ±0.6 |
|  |  | **31** | 53.13 ±5.23 | 0.92 ±0.27 | 8.25 ±2.10 | -2 ±0.66 |
|  |  | **41** | 51.32 ±4.53 | 0.49 ±0.24 | 12.04 ±3.31 | -1.28 ±0.6 |
|  |  | **56** | 57.76 ±7.75 | 0.72 ±0.42 | 23.59 ±2.99 | -1.32 ±0.74 |
|  |  | **81** | 53.99 ±4.29 | 1 ±0.44 | 24.98 ±5.73 | 1.04 ±0.41 |
|  | **1 way ANOVA (age)** | **F (4,41)** | 0.23 | 0.23 | 3.39 | 3.58 |
|  |  | **P** | 0.9209 | 0.918 | 0.0174 | 0.015 |
| **C3H** | **Age (weeks)** | **16** | 54.13 ±8.14 | 1.66 ±0.32 | 13.17 ±3.93 | -1.02 ±0.92 |
|  |  | **31** | 52.9 ±6.27 | 2.18 ±0.36 | 22.17 ±3.58 | -0.69 ±0.66 |
|  |  | **41** | 56.53 ±4.6 | 1.94 ±0.32 | 27.52 ±2.64 | -0.45 ±0.83 |
|  |  | **56** | 65.63 ±4.93 | 1.92 ±0.27 | 19.71 ±2.09 | -1.04 ±0.88 |
|  |  | **81** | 51.35 ±7.11 | 2.38 ±0.17 | 21.38 ±1.91 | 0.58 ±0.6 |
|  | **1 way ANOVA (age)** | **F (4,37)** | 0.79 | 0.63 | 3.27 | 0.66 |
|  |  | **P** | 0.5411 | 0.648 | 0.0215 | 0.625 |
| **C3PDE** | **Age (weeks)** | **16** | 59.47 ±7.78 | 1.77 ±0.26 | 23.19 ±4.88 | -0.96 ±0.8 |
|  |  | **31** | 62.95 ±4.25 | 1.77 ±0.36 | 27.67 ±3.16 | -0.33 ±0.77 |
|  |  | **41** | 54.88 ±6.38 | 2.3 ±0.29 | 25.66 ±2.85 | -0.29 ±0.54 |
|  |  | **56** | 53.31 ±3.93 | 1.95 ±0.22 | 27.34 ±3.94 | -0.77 ±0.67 |
|  |  | **81** | 50.91 ±8.06 | 1.75±0.29 | 27.38 ±3.41 | 0.46 ±0.68 |
|  | **1 way ANOVA (age)** | **F (4,34)** | 0.66 | 0.34 | 0.255 | 0.36 |
|  |  | **P** | 0.6249 | 0.847 | 0.9046 | 0.833 |
| **2-way ANOVA** | **Age** | **F (4,149)** | 0.634 | 1.127 | 3.114 | 2.616 |
|  |  | **P** | 0.639 | 0.348 | 0.017 | 0.038 |
|  | **Strain** | **F (3,149)** | 0.253 | 17.223 | 13.488 | 5.107 |
|  |  | **P** | 0.859 | ≤0.0000001 | ≤0.0000001 | 0.002 |
|  | **Age X Strain** | **F (12,149)** | 0.62 | 0.621 | 1.626 | 0.95 |
|  |  | **P** | 0.822 | 0.82 | 0.09 | 0.5 |

Table S7: Principal component analysis (PCA). PCA gives rise to a 10 component solution accounting for 81.7% of total variance. The variance accounted by each component is shown on the table below in brackets below the component number.

|  | **Component** | | | | | | | | | |
| --- | --- | --- | --- | --- | --- | --- | --- | --- | --- | --- |
|  | **1** | **2** | **3** | **4** | **5** | **6** | **7** | **8** | **9** | **10** |
|  | **(25.2%)** | **(12%)** | **(7.3%)** | **(6.5%)** | **(6.1%)** | **(5.9%)** | **(5.2%)** | **(4.8%)** | **(4.7%)** | **(4%)** |
| **Circadian activity in DD** | **0.912** |  |  |  |  |  |  |  |  |  |
| **Circadian activity in dark phase of LD** | **0.911** |  |  |  |  |  |  |  |  |  |
| **Total circadian activity in LD** | **0.908** |  |  |  |  |  |  |  |  |  |
| **Distance travelled in dark by video tracking** | **0.885** |  |  |  |  |  |  |  |  |  |
| **Speed of movement in dark by video tracking** | **0.885** |  |  |  |  |  |  |  |  |  |
| **Total distance travelled by video tracking** | **0.879** |  |  |  |  |  |  |  |  |  |
| **Speed of movement in light and dark by video tracking** | **0.879** |  |  |  |  |  |  |  |  |  |
| **Circadian amplitude in LD** | **0.752** |  |  |  |  |  |  |  |  |  |
| **Circadian amplitude in DD** | **0.741** |  |  |  |  |  |  | -0.454 |  |  |
| **Number of sleep episodes in dark** | **-0.633** |  | 0.458 |  | 0.419 |  |  |  |  |  |
| **Intradaily variability in DD** | **-0.542** |  | 0.390 |  |  |  |  | 0.534 |  |  |
| **Intradaily variability in LD** | **-0.513** |  | 0.303 |  |  |  |  | 0.497 |  |  |
| **Interdaily stability in LD** | **0.459** |  | 0.458 |  |  |  |  |  |  | 0.347 |
| **Length of active phase in LD** |  | **0.778** |  |  |  |  |  |  |  |  |
| **Phase angle of entrainment** |  | **-0.726** |  |  |  |  |  |  |  |  |
| **Speed of movement in light by video tracking** | 0.326 | **0.704** |  |  |  |  |  |  |  |  |
| **Length of active phase in DD** |  | **0.693** |  |  |  |  |  |  |  |  |
| **Distance travelled in light by video tracking** | 0.326 | **0.693** |  |  |  | -0.319 |  |  |  |  |
| **Optokinetic drum score** | 0.319 | **0.618** |  |  |  |  |  |  |  |  |
| **Circadian activity in light phase of LD** | 0.409 | **0.566** |  |  |  |  |  |  |  |  |
| **Number of sleep episodes following light pulse** |  |  | **0.824** |  |  |  |  |  |  |  |
| **Proportion of time asleep following light pulse** |  |  | **0.783** |  |  |  |  |  |  |  |
| **Initial stage PLR constriction rate** |  |  |  | **0.936** |  |  |  |  |  |  |
| **Late stage PLR constriction rate** |  |  |  | **-0.931** |  |  |  |  |  |  |
| **Proportion of time asleep in the dark phase** | -0.498 |  |  |  | **0.749** |  |  |  |  |  |
| **Total proportion of time asleep** | -0.448 |  |  |  | **0.691** |  | -0.318 |  |  |  |
| **Circadian period** |  | 0.347 | 0.344 |  | **-0.369** |  | 0.318 |  |  |  |
| **No of sleep episodes during light pulse** |  |  |  |  |  | **.808** |  |  |  |  |
| **Proportion of time asleep during light pulse** |  |  |  |  |  | **.708** |  |  |  |  |
| **Speed of sleep induction during light pulse** |  | -0.381 |  |  |  | **.504** |  |  |  | 0.332 |
| **No of sleep episodes during light phase** |  |  |  |  |  |  | **0.881** |  |  |  |
| **Total no of sleep episodes** | -0.549 |  | 0.371 |  |  |  | **0.558** |  |  |  |
| **Proportion of time asleep in the light phase** |  |  |  |  |  | .547 | **-0.552** |  |  |  |
| **Proportion of circadian activity in light phase** |  | 0.517 |  |  |  |  |  | **0.572** |  |  |
| **PLR maximum relative constriction** |  | -0.412 |  | -0.410 | 0.320 |  |  | **0.516** |  |  |
| **Speed of pupil recovery following light** |  |  |  |  |  |  |  |  | **-0.910** |  |
| **Speed of waking following light pulse** |  |  |  |  |  |  |  |  |  | **0.840** |
